# Improving Linkage to the HCV Care Cascade in Nonspecialist Settings: Evaluation of the Performance and Triage Utility of the Elecsys HCV Duo Assay in Japan

**DOI:** 10.1002/jcla.70286

**Published:** 2026-06-16

**Authors:** Hiroko Setoyama, Takehisa Watanabe, Shiho Miyase, Masakuni Tateyama, Motohiko Tanaka, Nobuhiro Minami, Masato Sasaki, Shunpei Hashigo, Kotaro Fukubayashi, Shotaro Ishii, Mutsuo Koga, Ryo Ichikawa, Hirofumi Iwashita, Yoshinari Sakai, Toshihiko Motohara, Kohei Tomita, Akinori Nakata, Yutaka Kai, Takeyasu Kounoe, Katsuki Haraoka, Takako Inoue, Takanori Suzuki, Kentaro Matsuura, Yasuhito Tanaka

**Affiliations:** ^1^ Department of Gastroenterology and Hepatology, Faculty of Life Sciences Kumamoto University Kumamoto Japan; ^2^ Department of Gastroenterology and Hepatology Kumamoto Shinto General Hospital Kumamoto Japan; ^3^ National Hospital Organization Kumamoto Medical Center Kumamoto Japan; ^4^ Saiseikai Kumamoto Hospital Kumamoto Japan; ^5^ Japanese Red Cross Kumamoto Hospital Kumamoto Japan; ^6^ Kumamoto Rosai Hospital Kumamoto Japan; ^7^ Kumamoto City Hospital Kumamoto Japan; ^8^ Kumamoto Kenhoku Hospital Kumamoto Japan; ^9^ Kumamoto General Hospital Kumamoto Japan; ^10^ JCHO Hitoyoshi Medical Center Kumamoto Japan; ^11^ Minamata City General Hospital and Medical Center Kumamoto Japan; ^12^ Kumamoto Regional Medical Center Kumamoto Japan; ^13^ Amakusa Medical Center Kumamoto Japan; ^14^ Yamaga City Medical Center Kumamoto Japan; ^15^ Kamiamakusa General Hospital Kumamoto Japan; ^16^ National Hospital Organization Kumamotominami Hospital Kumamoto Japan; ^17^ Aso Medical Center Kumamoto Japan; ^18^ Shinsei Midori Hospital Kumamoto Japan; ^19^ Kumamoto Chuo Hospital Kumamoto Japan; ^20^ Department of Clinical Laboratory Medicine Nagoya City University Hospital Nagoya Japan; ^21^ Department of Gastroenterology and Metabolism Nagoya City University Hospital Nagoya Japan

**Keywords:** Elecsys HCV Duo, HCV care cascade, HCV core antigen, hepatitis C virus, linkage to care, nonspecialist settings, screening strategy

## Abstract

**Background:**

In Japan, the hepatitis C virus (HCV) care cascade is constrained at the transition from anti‐HCV antibody screening to confirmatory HCV ribonucleic acid (HCV RNA) testing in nonspecialist departments. As HCV prevalence declines and the population ages, the two‐step antibody‐to‐RNA algorithm becomes less efficient and vulnerable to loss to follow‐up. We evaluated whether the Elecsys HCV Duo assay could function as a triage test to strengthen linkage to care.

**Methods:**

We conducted a two‐part study in Kumamoto Prefecture, Japan. First, a survey of 19 specialized institutions assessed hepatitis virus testing practices and downstream evaluation in nonspecialist departments. Second, 479 serum samples from patients undergoing HCV RNA quantification at four hospitals were prospectively evaluated using the Elecsys HCV Duo assay, with detection rates stratified by viral load.

**Results:**

Nonspecialist departments accounted for 86.6% of in‐hospital anti‐HCV testing; however, among 1416 anti‐HCV–positive individuals identified outside gastroenterology departments, only 22.7% underwent confirmatory HCV RNA testing. In the prospective evaluation of 479 samples, HCVcAg sensitivity and specificity versus HCV RNA were 62.8% and 99.8%, respectively. Detection rates increased with viral load, from 0% at ≤ 4.0 log_10_ IU/mL to 16.7% at 4.1–5.5 log_10_ IU/mL and 86.2% at ≥ 5.6 log_10_ IU/mL.

**Conclusion:**

A vulnerability in the HCV care cascade lies at the transition from screening to confirmatory evaluation in nonspecialist settings. When implemented as a triage test within referral pathways, the Elecsys HCV Duo assay may improve linkage to specialist care while reserving HCV RNA testing for downstream confirmation.

## Graphical abstract

In nonspecialist departments, most anti‐HCV–positive individuals do not proceed to RNA testing. The Elecsys HCV Duo assay simultaneously detects anti‐HCV antibodies and core antigen from a single blood draw and preferentially identifies patients with clinically meaningful viral loads. When used as an entry‐level triage test, this approach enables targeted referral and strengthens linkage to specialist care within the HCV care cascade.

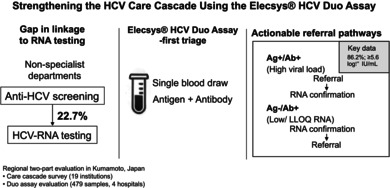

## 1 Introduction

Japan's hepatitis C virus (HCV) care cascade is increasingly constrained at a single critical step: the transition from anti‐HCV antibody (anti‐HCV) positivity to confirmatory RNA testing. As the infected population ages, many individuals are first identified in nonspecialist departments with normal alanine aminotransferase levels, and follow‐through to RNA testing declines. In parallel, RNA positivity among antibody‐positive individuals continues to decrease as prevalence falls, rendering the conventional two‐step (antibody → RNA) testing algorithm progressively less efficient and more vulnerable to loss to follow‐up [1].

Although HCV prevalence in Japan has steadily declined, the disease burden remains substantial. Analyses of the Japanese National Database have estimated that nearly 1 million people were living with chronic HCV infection in the mid‐2010s, with more than half either undiagnosed or not linked to care [2]. Despite the availability of highly effective direct‐acting antivirals (DAAs), ensuring timely linkage from diagnosis to treatment remains a central challenge, underscoring the need for screening strategies that are operationally robust and scalable within routine clinical practice [3]. Importantly, many individuals with chronic HCV infection still represent active HCV RNA‐positive cases requiring confirmatory testing and linkage to specialist care.

To address the vulnerable transition from screening to confirmation, a diagnostic approach that is simple, actionable, and deployable at first patient contact is required. The Elecsys HCV Duo assay meets this need by simultaneously detecting anti‐HCV antibodies and HCV core antigen (HCVcAg) from a single blood draw, enabling same‐visit triage without immediate reliance on RNA testing. Although HCVcAg assays are less sensitive than RNA at low‐level viremia, the HCV Duo assay demonstrates very high specificity and preferentially detects infections with higher viral loads [4, 5]. This performance profile aligns with the characteristics of most treatment‐eligible patients encountered at initial assessment and supports the use of antigen positivity as a trigger for expedited referral and downstream RNA confirmation within specialist care [6].

From an operational perspective, a Duo‐first strategy has the potential to reframe RNA testing from a prerequisite gatekeeper to a downstream confirmatory and quantitative step, thereby protecting the weakest link in the care cascade without compromising clinical accuracy where it matters most. When embedded within coordinated referral workflows, such an approach may accelerate linkage to hepatology services, reduce dependence on on‐site RNA testing in nonspecialist departments, and sustain case‐finding as seroprevalence and RNA positivity continue to decline [7].

Against this backdrop, we conducted a pragmatic, two‐part evaluation in Kumamoto Prefecture. First, we surveyed specialized institutions of liver disease to characterize testing practices in nonspecialist departments and to identify bottlenecks in the regional HCV care cascade. Second, we prospectively evaluated the performance of the Elecsys HCV Duo assay in routine clinical care, stratified by viral load, to determine where this assay provides the greatest operational yield. The primary objective of this study was to assess the feasibility and diagnostic yield of a Duo‐first strategy. We also sought to discuss referral pathways for antigen‐positive and antigen‐negative/antibody‐positive cases and to identify implementation metrics that should be evaluated in future scale‐up studies.

## 2 Materials and Methods

### 2.1 Survey of Specialized Institutions of Liver Disease in Kumamoto

In fiscal year 2025, a questionnaire‐based survey was conducted among all 19 specialized institutions of liver disease in Kumamoto Prefecture, Japan. The survey was designed to capture routine clinical practices during the preceding fiscal year (FY2024). Responses were obtained from all institutions (institutional response rate, 100%), although some survey items had incomplete responses.

As defined a priori, “nonspecialist departments” referred to hospital departments within specialized institutions of liver disease that do not specialize in hepatology. For these nonspecialist departments, the survey collected information on: (i) the proportion of hepatitis virus‐related laboratory testing among all in‐hospital laboratory tests; (ii) positivity rates for hepatitis B surface antigen (HBsAg) and anti‐HCV antibodies; and (iii) rates of subsequent evaluation among patients with positive test results, including confirmatory testing and/or referral to hepatology services, according to each institution's routine clinical pathways.

### 2.2 Evaluation of the Elecsys HCV Duo Assay

Between May and December 2024, a total of 479 consecutive serum samples were prospectively collected from patients undergoing HCV RNA quantification at four hospitals in Kumamoto Prefecture: Kumamoto University Hospital, Kumamoto Shinto General Hospital, National Hospital Organization Kumamoto Medical Center, and Saiseikai Kumamoto Hospital. All samples were obtained in the course of routine clinical care. The prospective assay cohort was independent of the survey cohort. Specifically, the 479 serum samples used for Elecsys HCV Duo evaluation were consecutively collected from patients undergoing HCV RNA quantification at four participating hospitals and were not derived from the 1416 anti‐HCV–positive individuals identified through the institutional survey.

Each sample was tested using the Elecsys HCV Duo assay (Roche Diagnostics GmbH, Mannheim, Germany), which simultaneously detects anti‐HCV antibodies and HCVcAg. The Elecsys HCV Duo assay is an electrochemiluminescence immunoassay in which HCVcAg and anti‐HCV are measured in two separate parallel reactions from a single specimen. Antibody detection employs a double‐antigen sandwich format using recombinant HCV antigens, whereas core antigen detection utilizes monoclonal antibodies directed against the HCV core protein.

The HCV Duo assay was performed on the cobas 8000 e801 module, and results were interpreted according to the manufacturer's instructions. Results were reported as a cutoff index (COI). Samples with COI < 1.0 were interpreted as nonreactive and those with COI ≥ 1.0 as reactive. According to the manufacturer's protocol, initially reactive samples were retested in duplicate. If both repeat measurements were < 1.0, the final result was considered nonreactive; if one or both repeat measurements were ≥ 1.0, the sample was considered repeatedly reactive and required confirmatory testing (e.g., HCV RNA testing). The analytical sensitivity for HCVcAg reported by the manufacturer is 50 IU/mL, standardized against the WHO International Standard for HCVcAg.

HCV RNA levels were measured using the COBAS AmpliPrep/COBAS TaqMan HCV Quantitative Test, version 2.0 (Roche Diagnostics GmbH), with a lower limit of quantification (LLOQ) of 1.2 log_10_ IU/mL. HCV RNA testing using this assay was used as the reference standard for calculating the diagnostic performance of the HCVcAg component of the Elecsys HCV Duo assay.

Statistical analyses were performed using IBM SPSS Statistics version 19.0 (IBM Corp., Armonk, NY, USA). Differences in HCV RNA levels between the Ag+/Ab+ and Ag−/Ab+ groups were evaluated using an independent‐samples Student's *t*‐test. A *p* value < 0.05 was considered statistically significant.

HCV testing and linkage‐to‐care practices in participating institutions generally followed international clinical guidelines, including those issued by the World Health Organization (WHO) [8] and other major hepatology societies.

For descriptive analysis, detection rates were stratified by HCV RNA viral load into three predefined categories: ≤ 4.0, 4.1–5.5, and ≥ 5.6 log_10_ IU/mL. Analyses comparing antigen‐positive and antigen‐negative groups were restricted to anti‐HCV–positive samples.

### 2.3 Handling of HCV RNA Values Below the Limit of Quantification

For graphical presentation, HCV RNA values below the LLOQ (1.2 log_10_ IU/mL) were treated as censored data. In box‐and‐whisker plots, such values were set to 1.2 log_10_ IU/mL for visualization purposes only.

## 3 Results

### 3.1 Survey of Specialized Institutions of Liver Disease

All 19 specialized institutions of liver disease in Kumamoto Prefecture responded to the survey (institutional response rate, 100%). Nonspecialist departments accounted for the majority of in‐hospital hepatitis virus‐related laboratory testing, representing 86.6% of all anti‐HCV tests performed during the study period.

**FIGURE 1:**
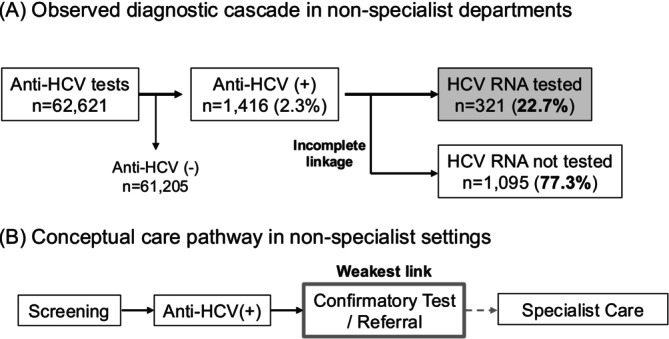
(A) Observed HCV diagnostic cascade in nonspecialist departments. Among individuals who tested positive for anti‐HCV antibodies, only 22.7% underwent confirmatory HCV RNA testing, indicating substantial loss to follow‐up after initial screening. These numbers were derived from the institutional survey cohort and do not represent the separate prospective serum‐sample cohort used for Elecsys HCV Duo assay evaluation. (B) Conceptual HCV care pathway in nonspecialist settings, highlighting the transition from antibody positivity to confirmatory testing and/or referral as the weakest link in the cascade.

Among anti‐HCV‐positive individuals identified in nonspecialist departments, only 22.7% proceeded to confirmatory HCV RNA testing, whereas the remaining 77.3% did not undergo confirmatory evaluation (Figure 1A). A similar pattern was observed among patients who tested positive for HBsAg.

This discrepancy between widespread screening and incomplete follow‐up indicates a gap between initial screening and confirmatory evaluation within the current hepatitis virus care cascade in nonspecialist departments (Figure 1B).

The data shown in Figure 1A represent the survey cohort at the institutional level and are distinct from the independent prospective serum‐sample cohort used for HCV Duo performance evaluation.

### 3.2 Performance of the Elecsys HCV Duo Assay

**FIGURE 2:**
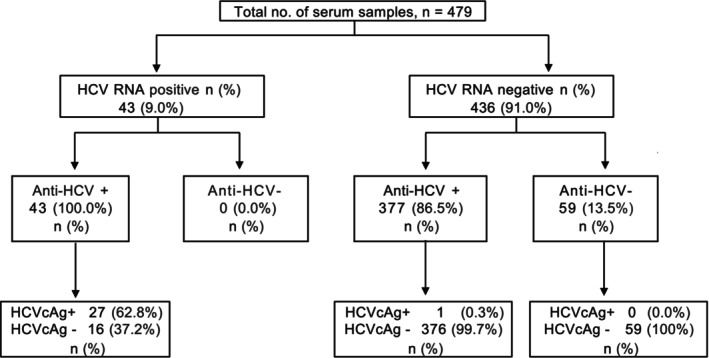
A total of 479 serum samples were classified according to HCV RNA status, anti‐HCV antibody reactivity, and HCV core antigen reactivity. Among 43 HCV RNA‐positive samples, 27 were HCVcAg positive and 16 were HCVcAg negative. Among 436 HCV RNA‐negative samples, one was HCVcAg positive and 435 were HCVcAg negative.

A total of 479 serum samples were tested using the Elecsys HCV Duo assay. Of these, *n* = 28 were Ag+/Ab+, *n* = 392 were Ag−/Ab+, *n* = 59 were Ag−/Ab−, and *n* = 0 were Ag+/Ab−. Using HCV RNA testing as the reference standard across all 479 prospectively evaluated samples, the HCVcAg component of the Elecsys HCV Duo assay was positive in 27 of 43 HCV RNA‐positive samples and negative in 435 of 436 HCV RNA‐negative samples, yielding an overall sensitivity of 62.8% and specificity of 99.8%. The classification of the 479 prospectively evaluated samples according to HCV RNA status, anti‐HCV antibody reactivity, and HCVcAg reactivity is summarized in Figure 2.

**FIGURE 3:**
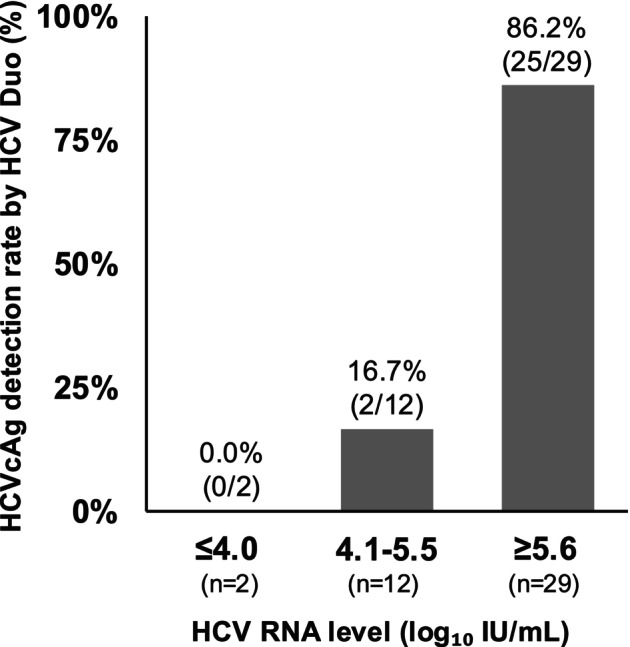
Detection rates of the Elecsys HCV Duo assay stratified by HCV RNA viral‐load category (≤ 4.0, 4.1–5.5, and ≥ 5.6 log_10_ IU/mL). Detection increased with higher viral load, with no detection observed at the lowest viral‐load stratum and the highest detection rate observed at ≥ 5.6 log_10_ IU/mL.

Detection rates varied markedly according to HCV RNA viral load. No samples with RNA levels ≤ 4.0 log_10_ IU/mL were detected by the HCV Duo assay (0/2), whereas detection rates were 16.7% for samples with RNA levels of 4.1–5.5 log_10_ IU/mL (2/12) and increased to 86.2% for samples with RNA levels ≥ 5.6 log_10_ IU/mL (25/29) (Figure 3). This pattern indicates preferential detection of cases with higher viral loads, which are most relevant for treatment prioritization and referral.

**FIGURE 4:**
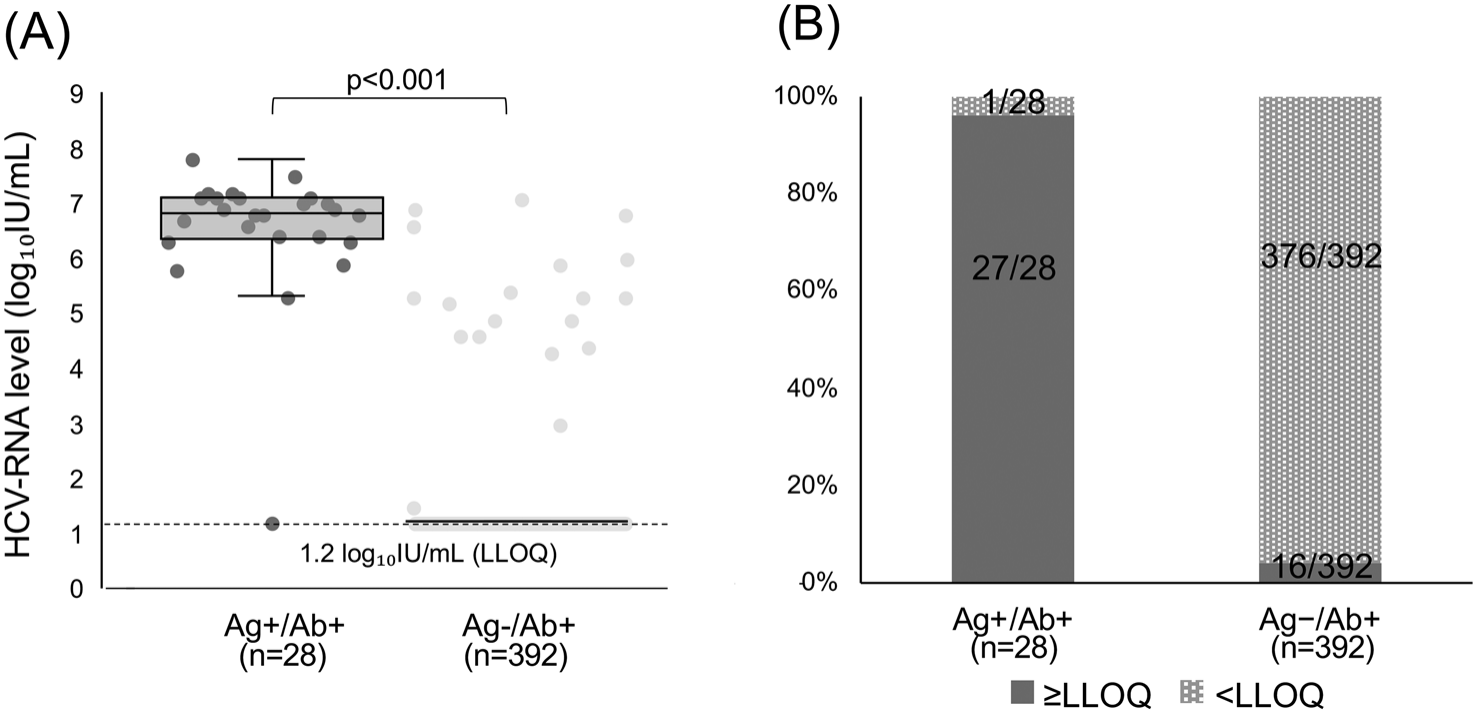
(A) Distribution of HCV RNA levels among anti‐HCV–positive patients who were positive for HCV antigen (Ag+/Ab+, *n* = 28) and those who were antigen‐negative (Ag−/Ab+, *n* = 392). RNA levels are shown on a log_10_ IU/mL scale. Values below the lower limit of quantification (LLOQ; 1.2 log_10_ IU/mL) were set to 1.2 log_10_ IU/mL for visualization. Boxes indicate the interquartile range, center lines indicate the median, and points represent individual samples. Differences between groups were evaluated using an independent‐samples *t*‐test (*p* < 0.001). (B) Proportion of anti‐HCV–positive samples with quantifiable HCV RNA (≥ LLOQ) and those below the LLOQ among Ag+/Ab+ (*n* = 28) and Ag−/Ab+ groups (*n* = 392). Quantifiable HCV RNA was observed in 27/28 (96.4%) of Ag+/Ab+ samples and 16/392 (4.1%) of Ag−/Ab+ samples; the remaining samples were below the LLOQ (1/28 and 376/392, respectively).

Patients who tested positive for both HCVcAg and anti‐HCV exhibited significantly higher HCV RNA levels than those who were antibody‐positive but antigen‐negative (*p* < 0.001) (Figure 4A). In contrast, antigen‐negative cases were predominantly characterized by low‐level viremia, with a substantial proportion of samples falling below the LLOQ. As shown in Figure 4B, quantifiable HCV RNA levels (≥ LLOQ) were present in 96.4% (27/28) of the Ag+/Ab+ group, whereas only 4.1% (16/392) of the Ag−/Ab+ group had quantifiable HCV RNA; conversely, most Ag−/Ab+ samples were below the LLOQ. Importantly, no instances of antigen positivity in the absence of antibody positivity were observed, supporting the high specificity and clinical reliability of the HCV Duo assay in routine practice.

## 4 Discussion

This study provides proof of concept for a pragmatic, cascade‐oriented diagnostic strategy to address a persistent vulnerability in HCV care: the failure to complete confirmatory evaluation after anti‐HCV positivity in nonspecialist departments. Although nonspecialist services account for the majority of anti‐HCV testing and identification of antibody‐positive individuals, fewer than one‐quarter of patients identified outside gastroenterology services proceeded to HCV RNA testing. This finding highlights a structural weakness in routine clinical practice, whereby opportunities for case detection are widely available, but linkage to definitive evaluation and specialist care remain inconsistent.

The operational value of antigen‐based assays is intrinsically influenced by viral load, a factor that is central to their role in simplified diagnostic pathways. In this study, detection by the Elecsys HCV Duo assay showed a clear dependence on HCV RNA levels, with substantially higher detection rates at clinically meaningful viral loads. Patients who were positive for both HCVcAg and anti‐HCV consistently exhibited higher RNA levels than those who were antibody‐positive but antigen‐negative, while the latter group was frequently characterized by low or unquantifiable viremia. Some samples with viral loads > 4.1 log_10_ IU/mL remained antigen‐negative. This finding is consistent with the known limitation of HCVcAg assays, whose sensitivity decreases in samples with low‐level viremia. This may reflect the lower analytical sensitivity of the antigen component of the HCV Duo assay compared with dedicated standalone HCV core antigen chemiluminescent microparticle immunoassay (CMIA) platforms. Although previous studies and meta‐analyses have reported generally high sensitivities for HCVcAg assays, including in people living with HIV, the reported sensitivity varies according to the assay platform, study population, and distribution of viral loads [9]. Studies including immunocompromised populations, such as patients with chronic kidney disease, hemodialysis, or pre/posttransplant status, have reported sensitivities ranging approximately from 80% to 95%, with reduced sensitivity most evident in samples with low HCV RNA levels, especially below approximately 10^3^ IU/mL [10, 11]. Therefore, antibody‐positive but antigen‐negative results should not be interpreted as excluding active infection, and reflex RNA testing or predefined follow‐up pathways remain essential.

These findings indicate that antigen positivity reliably identifies individuals who are more likely to have higher viral loads and may benefit from timely referral to specialist care, consistent with previous reports on the clinical utility of HCV antigen–antibody combination testing in Japan [12]. The reduced sensitivity of HCVcAg observed in several standalone CMIA‐based assays and HCV Duo antigen–antibody combination assays represents an important research gap and clinical challenge in HCV diagnosis. To address this limitation, strategies to improve HCVcAg sensitivity should be explored, including further investigation of the dissociation characteristics and kinetics of HCVcAg in immune complexes with anti‐core antibodies. Such improvements may facilitate immediate linkage to care, particularly among patients with low‐level viremia or immunocompromised conditions, and may contribute to achieving the WHO goal of HCV elimination by 2030 in the era of highly effective pan‐genotypic direct‐acting antivirals.

The observed diagnostic gap must be interpreted in the context of evolving HCV epidemiology. As HCV seroprevalence declines and the infected population ages, the widespread use of highly effective DAAs has resulted in very high rates of sustained virologic response. Consequently, the proportion of RNA‐positive individuals among those who are antibody‐positive has decreased. In nonspecialist settings, this epidemiological shift may contribute to the perception that anti‐HCV positivity does not necessarily indicate active infection, thereby reducing the perceived urgency of RNA‐based confirmatory testing. Although antibody screening remains widely implemented, downstream confirmatory evaluation and referral often require additional coordination and are not consistently completed.

Within this contemporary landscape, the HCV Duo assay may serve a clearly defined operational role as an entry‐level triage tool at first patient contact. Antigen positivity provides an immediately actionable signal that can prompt expedited referral to hepatology services, where confirmatory and quantitative RNA testing can be performed under conditions that ensure continuity of care. By suggesting a potential repositioning of RNA testing from an initial gatekeeping requirement in nonspecialist departments to a downstream confirmatory step within specialist care, this approach may better align diagnostic sequencing with real‐world clinical workflows and help strengthen continuity along the care cascade.

Importantly, antibody‐positive but antigen‐negative results should not be interpreted as excluding active infection. For this subgroup, structured safeguards—such as reflex RNA testing or predefined follow‐up pathways—are essential to prevent missed diagnoses and inappropriate reassurance. Accordingly, the effectiveness of a Duo‐first strategy depends not solely on assay performance but on its integration into clearly defined referral pathways and care systems that ensure completion of the diagnostic process.

Several limitations warrant consideration. This study was conducted within a single prefecture, and the prospective evaluation was limited to four hospitals, which may limit generalizability to primary‐care or community‐based settings. Reduced sensitivity at low viral loads is an inherent limitation of core antigen‐based assays. In addition, cost‐effectiveness, time to treatment initiation, and patient‐level clinical outcomes were not evaluated and should be addressed in future implementation studies.

In conclusion, this study demonstrates that within a regional clinical network in Japan, nonspecialist departments perform most HCV testing, yet confirmatory evaluation is frequently not completed, representing a key vulnerability in the HCV care cascade. The Elecsys HCV Duo assay showed high specificity and preferential detection at clinically meaningful viral loads, supporting its role as an entry‐level triage test. When implemented within clearly defined referral pathways, a Duo‐first strategy offers a positive proof of concept for strengthening linkage to specialist care while reserving RNA testing for targeted downstream confirmation.

## Author Contributions

Study concept and design: Hiroko Setoyama and Yasuhito Tanaka. Data collection: Hiroko Setoyama, Takehisa Watanabe, Shiho Miyase, Masakuni Tateyama, Motohiko Tanaka, Nobuhiro Minami, Masato Sasaki, Shunpei Hashigo, Kotaro Fukubayashi, Shotaro Ishii, Mutsuo Koga, Ryo Ichikawa, Hirofumi Iwashita, Yoshinari Sakai, Toshihiko Motohara, Kohei Tomita, Yutaka Kai, Takeyasu Kounoe, Katsuki Haraoka, and Akinori Nakata. Data analysis and interpretation: Hiroko Setoyama and Yasuhito Tanaka. Manuscript drafting: Hiroko Setoyama and Yasuhito Tanaka. Critical revision of the manuscript: All authors. Final approval of the manuscript: All authors.

## Funding

This work was supported by a Grant‐in‐Aid from the Research Program on Hepatitis from the Japan Agency for Medical Research and Development (AMED; JP25fk0210172) and by research funding from Roche Diagnostics.

## Ethics Statement

This study was conducted in accordance with the principles of the Declaration of Helsinki and was approved by the Ethics Committee for Epidemiology and General Research, Graduate School of Medical Sciences, Kumamoto University (Approval No. 3046). The questionnaire survey and the prospective evaluation of serum samples were reviewed and approved as minimal‐risk observational research.

## Consent

For the prospective evaluation of the Elecsys HCV Duo assay, written informed consent was obtained from participants whenever required by institutional policy. In institutions where written consent was waived, an opt‐out consent procedure was implemented in accordance with local ethical regulations, and information about the study was made publicly available to allow patients to decline participation. The questionnaire survey collected institutional‐level data only and did not involve identifiable patient information; therefore, individual informed consent was not required.

## Conflicts of Interest

Yasuhito Tanaka has received scholarship donations from AbbVie GK and Otsuka Pharmaceutical Co. Ltd., research funding from AbbVie GK, FUJIREBIO Inc., Sysmex Corporation, GlaxoSmithKline PLC, Gilead Sciences Inc., and AstraZeneca, and lecture fees from AbbVie GK, Gilead Sciences Inc., Chugai Pharmaceutical Co. Ltd., ASKA Pharmaceutical Holdings Co. Ltd., GlaxoSmithKline PLC, AstraZeneca, Eisai, and HU Frontier within the past 36 months. Takako Inoue has received consigned and joint research funding from FUJIREBIO Inc., Sysmex Corporation. All other authors declare no conflicts of interest. This study was conducted under a research contract with Roche Diagnostics, which provided study materials. Roche Diagnostics had no role in the study design, data collection, analysis, interpretation of the results, or manuscript preparation.

## Data Availability

The data that support the findings of this study are available from the corresponding author upon reasonable request.
